# Therapeutic efficacy of rifalazil (KRM-1648) in a *M*. *ulcerans*-induced Buruli ulcer mouse model

**DOI:** 10.1371/journal.pone.0274742

**Published:** 2022-10-06

**Authors:** Hanako Fukano, Kazue Nakanaga, Masamichi Goto, Mitsunori Yoshida, Norihisa Ishii, Yoshihiko Hoshino

**Affiliations:** 1 Department of Mycobacteriology, Leprosy Research Center, National Institute of Infectious Diseases, Tokyo, Japan; 2 Department of Pathology, Field of Oncology, Kagoshima University Graduate School of Medical and Dental Sciences, Kagoshima, Japan; 3 National Sanatorium Tamazenshoen, Tokyo, Japan; Institut de Pharmacologie et de Biologie Structurale, FRANCE

## Abstract

Buruli ulcer (BU) is a skin disease caused by *Mycobacterium ulcerans* infection that requires long-term antibiotic treatment and/or surgical excision. In this study, we investigated the therapeutic efficacy of the rifamycin derivative, rifalazil (RLZ) (also known as KRM-1648), in an advanced *M*. *ulcerans* infection model. Six-week-old female BALB/c mice were infected with 3.25 x 10^4^ colony-forming units (CFU) of *M*. *ulcerans* subcutaneously into the bilateral hind footpads. At 33 days post-infection, when the footpads exhibited significant redness and swelling, mice were treated orally with 5 or 10 mg/kg of RLZ for up to 15 weeks. Mice were followed for an additional 15 weeks following treatment cessation. Untreated mice exhibited a progressive increase in footpad redness, swelling, and erosion over time, and all untreated mice reached to endpoint within 5–8 weeks post-bacterial injection. In the RLZ-treated mice, footpad redness and swelling and general condition improved or completely healed, and no recurrence occurred following treatment cessation. After 3 weeks of treatment, the CFU counts from the footpads of recovered RLZ-treated mice showed a 10^4^ decrease compared with those of untreated mice. We observed a further reduction in CFU counts to the detection limit following 6 to 15 weeks of treatment, which did not increase 15 weeks after discontinuing the treatment. Histopathologically, bacteria in the treated mice became fragmented one week after RLZ-treatment. At the final point of the experiment, all the treated mice (5mg/kg/day; n = 6, 10mg/kg/day; n = 7) survived and had no signs of *M*. *ulcerans* infection. These results indicate that the rifamycin analogue, RLZ, is efficacious in the treatment of an advanced *M*. *ulcerans* infection mouse model.

## Introduction

Buruli ulcer (BU) is an emerging chronic skin disease characterized by massive skin ulceration and persistent necrotic change, and is one of twenty neglected tropical diseases as defined by the World Health Organization (WHO) [1]. *Mycobacterium ulcerans* is the causative agent of BU and has been reported in more than 30 countries worldwide, particularly in tropical or subtropical areas, such as West Africa and the temperate zone of Australia [2, 3]. *M*. *ulcerans* produces a lipid-like exotoxin called mycolactone, which is encoded by the giant plasmid pMUM001 [4, 5]. Mycolactone inhibits cytokine synthesis inhibiting the translocation of proteins, including cytokines, to the endoplasmic reticulum targeting Sec61 [6]. In addition, recent study demonstrates that, extra cellular vesicles containing mycolactone induce an uncontrolled inflammation (targeting IL1B) promoting tissue destruction [7–10]. BU is classified into three categories to reflect lesion severity: category I, single small lesion; category II, non-ulcerative and ulcerative plaque and oedematous forms; and category III, disseminated and mixed forms, such as osteitis, osteomyelitis, and joint involvement [1]. As the disease progresses, skin grafting becomes necessary and leaves a lasting disability. Contamination of broken skin in aquatic environments seems to be one of the major risk factors for developing BU, but there are currently no effective preventative measures [11]. In addition, vaccine development has not progressed to date. Under the current circumstances, early diagnosis and appropriate antimicrobial treatments are essential to managing *M*. *ulcerans* infection. Currently, the WHO Technical Advisory Group on BU recommends an 8-week course of combination therapy of rifampin (10mg/kg once daily) and clarithromycin (7.5 mg/kg twice daily) or streptomycin (15mg/kg once daily). However, recent studies have demonstrated that rifampin induces cytochrome P450 (CYP450) 3A4, which decreases the blood concentration of clarithromycin [12, 13]. This suggests that the combination of clarithromycin and rifampin is not ideal for the treatment of BU, demonstrating a need for more potent or additional therapeutic options.

Rifalazil (RLZ) (also known as KRM-1648 and AMI-1648), 3’-hydroxy-5’-(4-isobutyl-1 piperazinyl) benzoxazinorifamycin, is a rifamycin analogue that blocks the β-subunit in RNA polymerase of bacteria, including mycobacterial species. Previously, we demonstrated *in vitro* and *in vivo* that RLZ had greater antimicrobial activity and inhibitory effect against *M*. *ulcerans* infection compared to rifampin [14]. In this present study, we evaluated the therapeutic efficacy of RLZ in an advanced murine *M*. *ulcerans* infection model.

## Materials and methods

### Test drug

Rifalazil (RLZ), 3’-hydroxy-5’-(4-isobutyl-1-piperazinyl) benzoxazinorifamycin was provided by Kaneka Corporation (Takasago, Hyogo, Japan) and was suspended in 2.5% gum Arabic-0.01% Tween 80 for oral administration.

### *M*. *ulcerans* bacterial suspension

*M*. *ulcerans* strain MU-4 (original name: ITM 97–107), which was isolated from a BU patient in Benin, was provided by Prof. Francoise Portaels (Institute of Tropical Medicine, Antwerp, Belgium). Bacteria were cultured at 32°C in Middlebrook 7H9 broth supplemented with 0.2% glycerol and 10% OADC enrichment for 14 days in the logarithmic phase of bacterial growth. The bacterial suspension was diluted with 7H9 broth, and the density was adjusted to 0.2 at 540 nm. Twenty-five microliters of bacterial suspension (3.25×10^4^ CFU) was used for injection. The number of bacterial cells (colony-forming units (CFU)) was determined by inoculating the suspension on Middlebrook 7H10 agar medium supplemented with 0.2% glycerol and 10% OADC Enrichment.

Uninfected control mice were inoculated with the same volume of 7H9 broth without bacterium.

### Experimental infection

Six-week-old female BALB/c mice were infected with 3.25×10^4^ CFU *M*. *ulcerans* in the bilateral hind footpads. Uninfected control mice (n = 5) received the same volume of 7H9 broth. At 33 days post-infection, when the infection is fully established as previously described, mice were treated with 5 or 10mg/kg of RLZ by oral gavage five days per week for up to 15 weeks (n = 41). Untreated control mice (n = 14) were infected with *M*. *ulcerans* but did not receive treatment. At 1, 3, 6, 9, 12, or 15 weeks post-infection, or at the time of sacrifice, mice were observed for gross footpad skin lesions, redness, and swelling by a venire caliper. The number of mice measured for footpad swelling is shown in S1 Table. Treatment was stopped at 15 weeks to observe bacterial recurrence. All procedures followed the regulations of the Animal Care and Use Committee of the National Institute of Infectious Diseases, Japan (Approval #k0004).

A humane endpoint by an International Council for Laboratory Animal Science (ICLAS) was adopted in our institute. The mice were euthanized (before the time of death) as soon as possible when they reached to a humane endpoint (piloerection and loss of movement). The animal health and behavior of mice were checked at least twice a week.

### CFUs

For CFU enumeration, 4–5 mice were sacrificed on weeks 1, 3, 6, 9, and 15 post-infection. The surface of lateral hind footpads were decontaminated with povidone-iodine and rinsed with PBS (−), and then homogenized in 5mL PBS (−) in a glass homogenizer. The resultant homogenate was subjected to a 10-fold serial dilution with PBS, and 100uL was inoculated on 7H10 agar. The number of CFUs was counted after an 8-week incubation at 32°C.

### Histopathology

Twelve animals in total (day 33, *n* = 2; day 39, *n* = 2; day 40, *n* = 2; day 42, *n* = 1; day 49, *n* = 1; day 54, *n* = 3; day 57, *n* = 1) were sacrificed for histopathological examination. After induction of deep anesthesia, perfusion fixation was performed using 10% buffered formalin. Hind limbs and general organs were embedded in paraffin, cut into 4-μm sections, and histopathologically examined by hematoxylin and eosin (H&E) and Fite-Faraco acid-fast staining.

### THP-1 macrophage infection

To compare the intracellular activity and synergistic efficacy with Streptomycin of RLZ and RFP, human THP-1 monocyte infection experiments, and imaging analysis was carried out by the High Content Imaging System. The human THP-1 monocytes (Riken: RCB3686) were routinely cultured in RPMI 1640 medium (WAKO) supplemented with 10 fetal bovine serum (Thermo Scientific) and Ampicillin (50μg/mL) at 37˚C and 5% CO_2_. 2 x 10^4^ cells/well were seeded into a Cell Carrier-96well Ultra microplate (Perkin Elmer) plates and differentiated to macrophage with PMA(10ng/mL) for 72h. The single monolayer THP-1 macrophages were infected by strain MU-4 at the multiplicity of infection (MOI) of 20, centrifuged at 1,000 rpm for 5 minutes, and incubated for 24 hours at 30˚C and 5% CO_2_. To exclude extracellular bacteria, wells were washed with PBS (-) and incubated with RPMI 1640 added with RFP10μg/mL, RFP10μg/mL+SM10μg/mL, RLZ 10μg/mL and RLZ 10μg/mL+SM10μg/mL for 72 hours at 30˚C and 5% CO_2_. After that, infected cells were fixed with 4%(v/v) paraformaldehyde (PFA) for 10 min at RT and then washed 3x with cold PBS and treated with 0.1% Triton X-100 for 10min, and then washed x3 with PBS. For immunostaining of intracellular mycobacteria, the cells were blocked in blocking buffer (1%[w/v] BSA + 22.52mg/mL glycine + 0.1%[w/v] Tween 20/PBS) for 30 min at RT and incubated primary with mouse anti-mycobacteria IgM (LAM antibody TMDU3)(MBL) (1:100) for 1h at RT in the dark chamber. The cells were washed x3 PBS and secondary goat anti-mouse IgG Alexa Fluor 488 (ab150113)(abcam) (1:1000) for 1h at RT in a dark chamber and washed x3 with PBS(-). For imaging nuclei and cytoplasm of THP-1, the cells were stained with Hoechst 33258 (Dojindo) (1:1000) and HCS Cell Mask Deep Red (Thermo Scientific) (1:20000) for 25min at RT and then washed x3 PBS(-). Stained cell images were acquired by using the High-Content Imaging System Operetta CLS (Perkin Elmer), with 20x Air/0.6 NA. The sum of fluorescence intensity (Alexa Flour 488) of the intracellular bacterium was automatically calculated by Harmony software (Perkin Elmer). All measurements were performed in sextuplicate. Obtained data were expressed as the mean ± the standard deviation (SD) and the statistical analysis was performed with GraphPad Prism 9. The statistical significance was calculated using the unpaired t-test, and the values of P<0.05 were considered to be significant.

## Results

All infected mice exhibited enhanced hind footpad redness and swelling starting at day 33. In the untreated control mice, hind footpad redness and swelling increased over time, and all untreated mice were reached to endpoint within 5–8 weeks post-bacterial injection.

**Fig 1:**
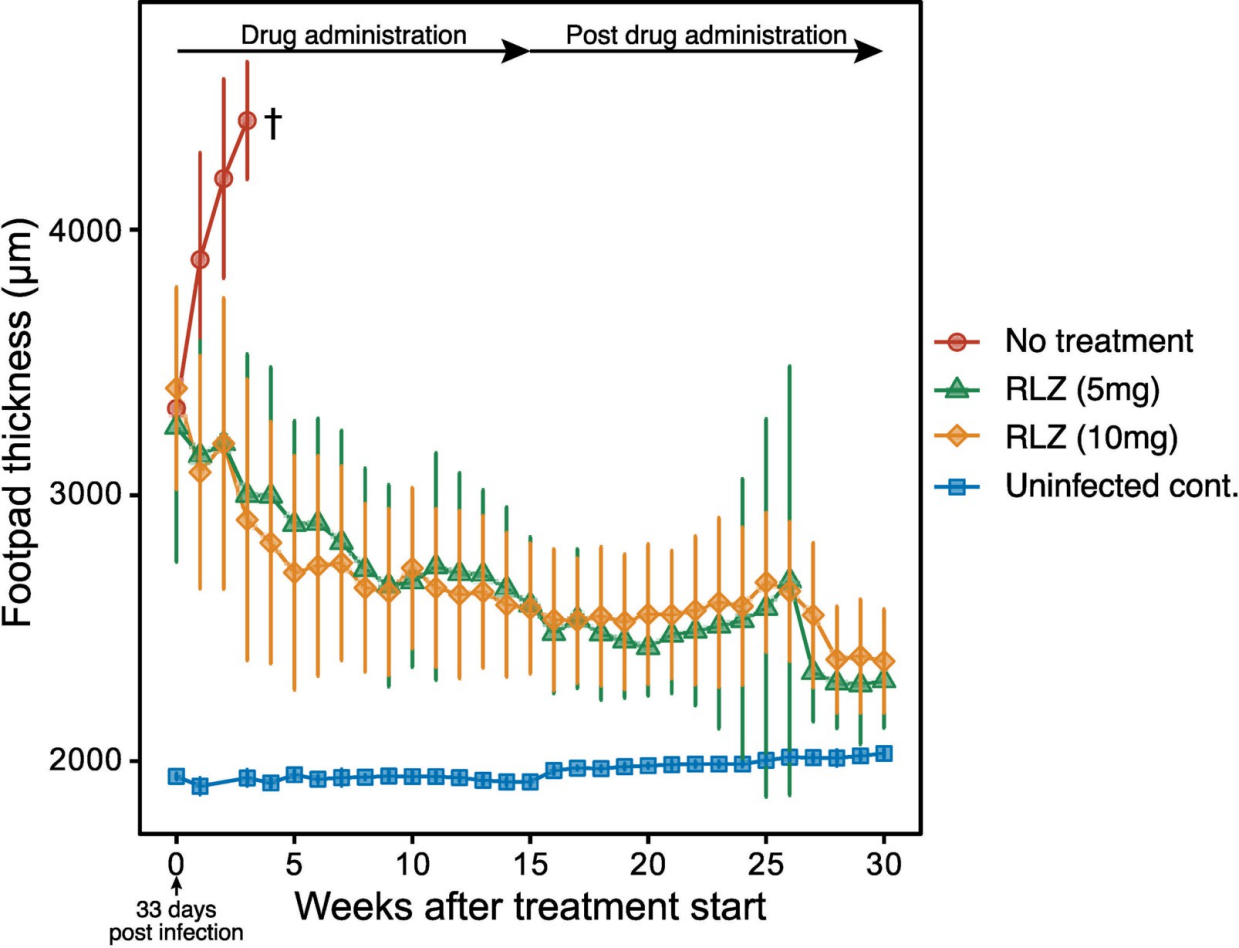
Successive measurement of hind footpad thickness. Measurements were started 33 days post-infection. † All of the mice in the untreated group reached to the endpoint at 4 weeks after the measurement was started.

In contrast, treatment with 5 mg/kg (n = 6) or 10 mg/kg (n = 7) of RLZ starting at day 33 improved or healed hind footpad lesions and redness and completely healed erosion 6 weeks after initiating treatment (Tables 1 and 2). In addition, both doses of RLZ significantly reduced footpad thickness, and swelling did not reoccur within 15 weeks following treatment cessation (Fig 1). All of the RLZ-treated mice survived the experiment course and were almost completely healed 30 weeks post-infection.

**Table 1:** Gross skin reddening of hind footpads in untreated and rifalazil-treated *M*. *ulcerans* infected mice.

	Drug administration							Post drug administration					
	0	1	3	6	9	12	15	1	3	6	9	12	15 (weeks)
Infected Control	14/14 ^a^	14/14	2/2										
	100% ^b^	100%	100%										
	1–2+ ^c^	2+	2+										
RLZ 5mg/kg/day	38/41	41/41	34/34	26/27	16/20	9/13	6/13	2/7	2/7	2/7	2/7	2/6	1/6
	100%	100%	100%	96.30%	80%	69.20%	46.20%	28.60%	28.60%	28.60%	28.60%	33.30%	16.70%
	1–2+	1–2+	1–2+	1+	1+	1+	1+	1+	1+	1+	1+	1+	1+
RLZ 10mg/kg/day	41/41	41/41	33/34	24/27	19/20	9/13	6/13	2/7	2/7	3/7	3/7	2/7	0/7
	100%	100%	97.1%	88.9%	95%	69.2%	46.2%	28.6%	28.6%	42.9%	42.9%	28.6%	0%
	1–2+	1–2+	1–2+	1+	1+	1+	1+	1+	1+	1+	1+	1+	-

a) Numerator indicates the number of mice exhibiting redness in one or both hind footpads and the denominator indicates the number of mice tested.

b) Percentages of mice exhibiting redness.

c) Degree of redness: 1+, slightly red; 2+, red

**Table 2:** Gross skin erosion of hind footpads in untreated and rifalazil-treated *M*. *ulcerans* infected mice.

	Drug administration							Post drug administration					
	0	1	3	6	9	12	15	1	3	6	9	12	15 (weeks)
Infected Control	0/14 ^a^	9/14	2/2										
	0% ^b^	64.3%	100%										
	- ^c^	1–2+	2–3+										
RLZ 5mg/kg/day	38/41	25/41	2/34	0/27	0/20	0/13	0/13	0/7	0/7	0/7	0/7	0/6	0/6
	0%	61%	5.9%	0%	0%	0%	0%	0%	0%	0%	0%	0%	0%
	-	1–2+	1+	-	-	-	-	-	-	-	-	-	-
RLZ 10mg/kg/day	0/41	27/41	0/34	0/27	0/20	0/13	0/13	0/7	0/7	0/7	0/7	0/7	0/7
	0%	65.9%	0%	0%	0%	0%	0%	0%	0%	0%	0%	0%	0%
	-	1+	-	-	-	-	-	-	-	-	-	-	-

a) Numerator indicates the number of mice exhibiting erosion in one or both hind footpads and denominator indicates the number of mice tested.

b) Percentages of mice exhibiting erosion.

c) Degree of erosion: -, none; 1+, partial; 2+, moderate; 3+, severe.

**Fig 2:**
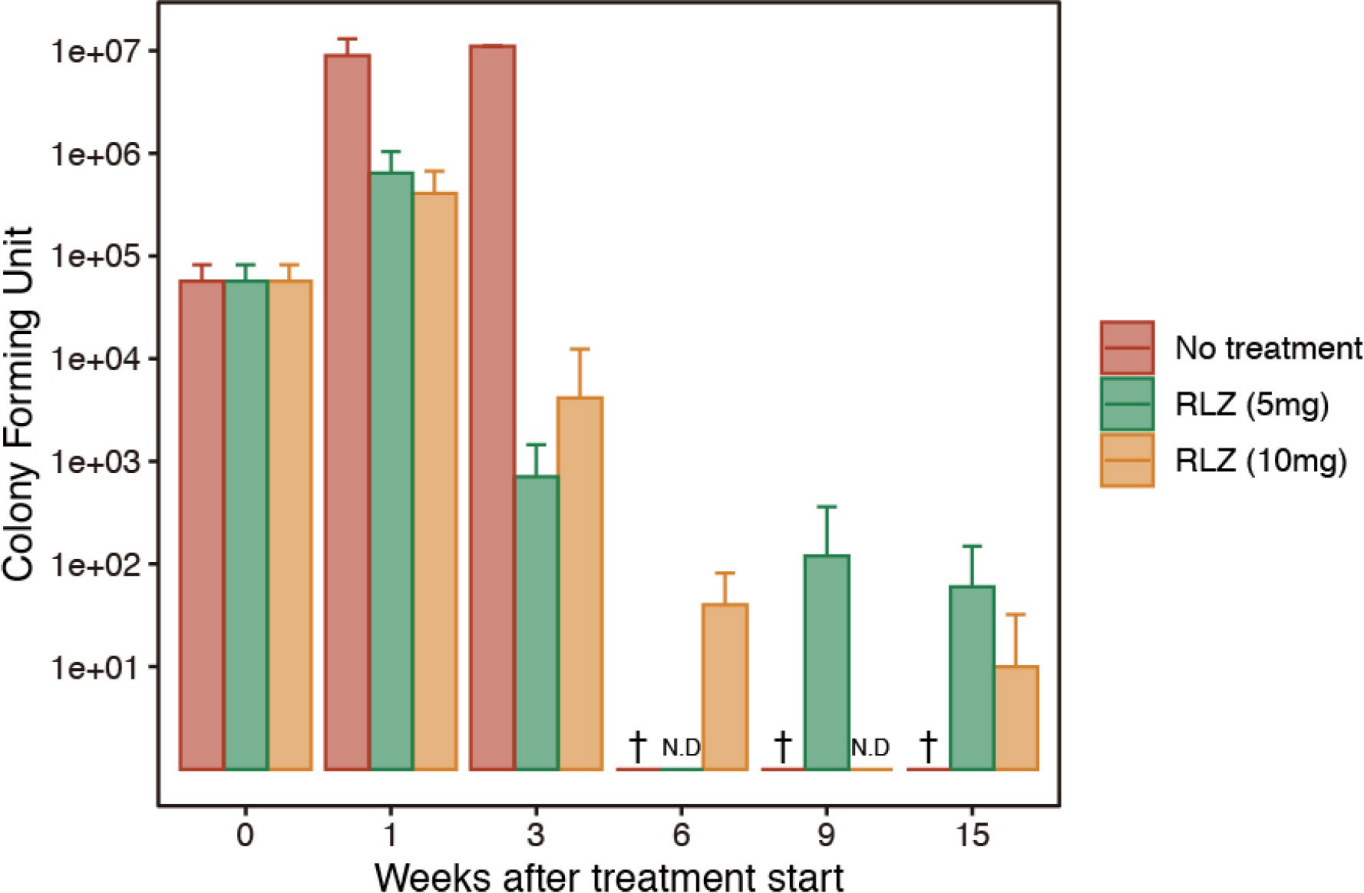
Log CFU numbers in the hind footpads. † All of the mice in the untreated group reached to the endpoint at 4 weeks after the measurement was started. N.D Colony was not detected.

After 3 weeks of treatment, mice treated with 5 and 10mg/kg of RLZ demonstrated an approximate 4-log (10^4^) decrease in the *M*. *ulcerans* CFU counts in both hind footpads compared with untreated mice (Fig 2). In RLZ-treated mice, CFU counts further decreased to the detection limit after 6 to 15 weeks of treatment and did not increase for 15 weeks after treatment cessation (Fig 2).

**Fig 3:**
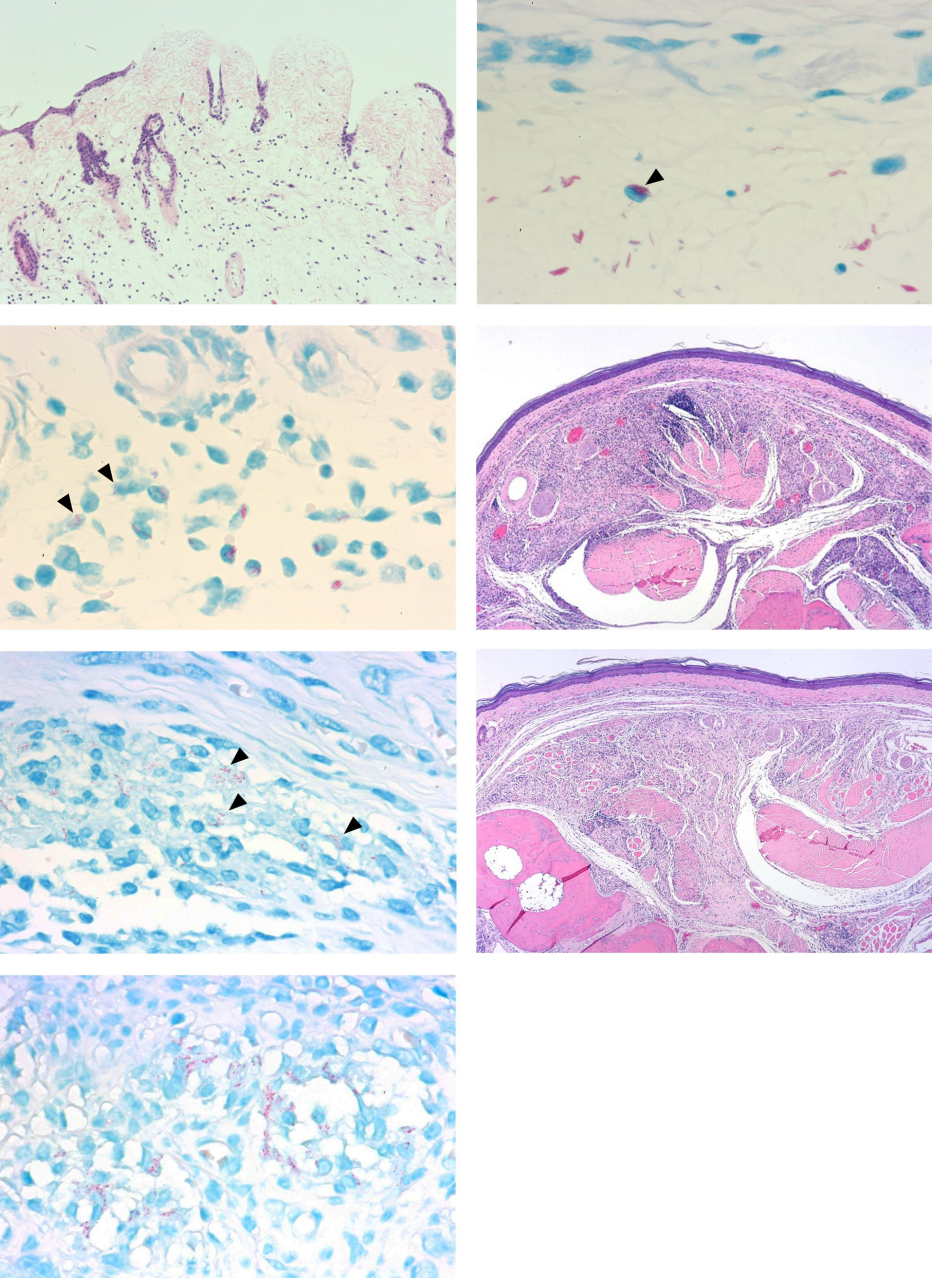
Histopathological features of hind footpads. A-B) Untreated control mouse at day 33 post-infection. A) Erosion, edema, and fibrinous exudates are observed (H&E x290). B) Aggregated acid-fast bacilli are observed primarily in the stroma, with some present in monocytes (arrowhead) (Fite-Faraco Staining x1750). C) Rifalazil (5mg/kg)-treated mouse at one-week of treatment. Some bacterial cells are fragmented (arrowheads) (Fite-Faraco staining x1750). D-E) Rifalazil (5mg/kg)-treated mouse at 15-weeks of treatment. D) Epithelioid cell granuloma with mild infiltration of lymphocytes is noted. Epidermal erosion, edema, fibrin, and neutrophil aggregation are not observed (H&E x120). E) Granularly degenerated acid-fast bacilli are observed in monocytes (arrowheads) (Fite-Faraco staining x1750). F-G) Rifalazil (5mg/kg)-treated mouse 15 weeks following termination of treatment. F) Epithelioid cell granuloma with mild infiltration of lymphocytes is noted. Epidermal erosion, edema, fibrin, and neutrophil aggregation are not observed (H&E x290). G) Completely degenerated acid-fast bacilli are observed in an epithelioid cell granuloma (Fite-Faraco staining x1750).

Histopathological analysis demonstrated ulcer, edema, and fibrinous exudates in untreated mice (Fig 3A). Aggregated solid acid-fast bacteria were located primarily in the stroma, with some identified in monocytes (Fig 3B). In RLZ-treated mice, the bacterial cells appeared fragmented after one week of treatment (Fig 3C). After 3 to 9 weeks of treatment, RLZ-treated mice exhibited healed erosion, absorption of stromal edema and fibrinous exudates, and epithelioid cell granuloma. At 15 weeks of treatment, epithelioid cell granulomas with mild infiltration of lymphocytes were present, without epithelial erosion, edema, fibrin, or neutrophil infiltration (Fig 3D). Small numbers of granularly degenerated acid-fast bacilli were observed in monocytes at this timepoint (Fig 3E). Similar histopathologic and bacteriologic findings were observed 15 weeks following cessation of RLZ treatment, indicating the absence of bacterial recurrence (Fig 3F). There were no significant differences in the macroscopic, bacteriologic, and histopathologic findings between mice treated with 5 or 10 mg/kg of RLZ.

**Fig 4:**
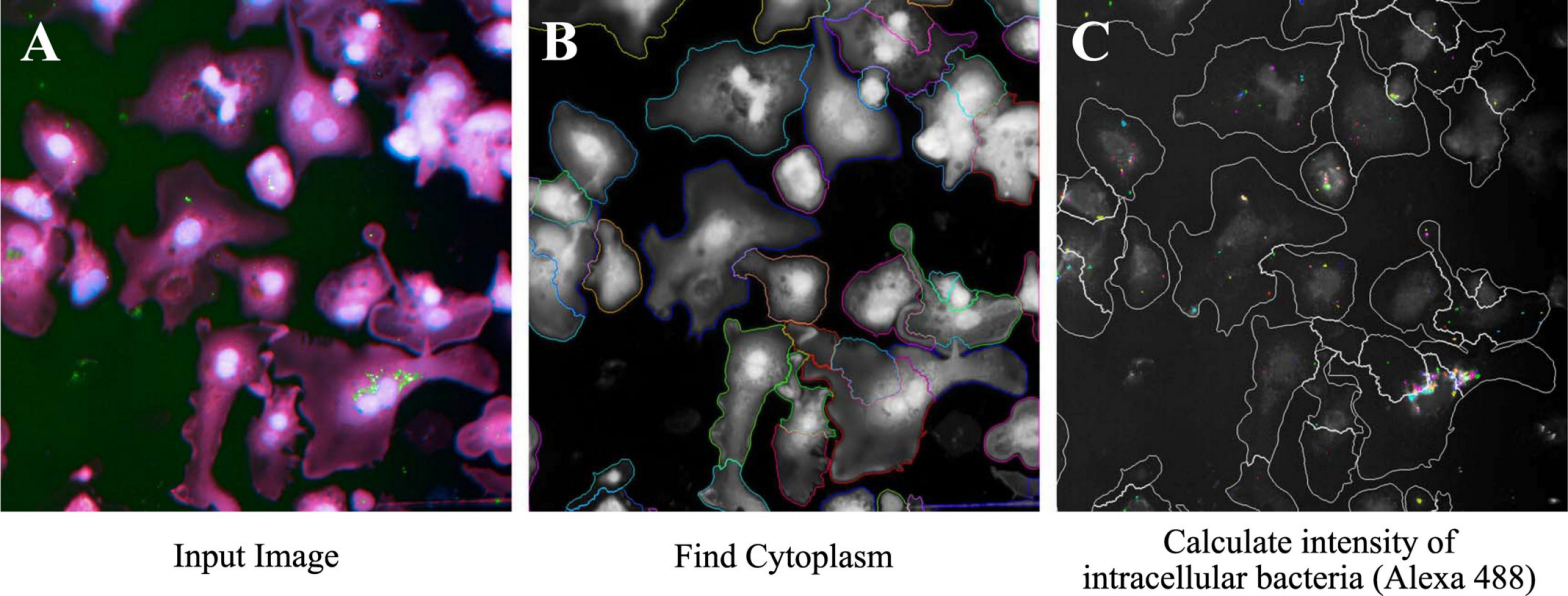
Workflow of Harmony software image analysis for intracellular *M*. *ulcerans*. THP-1 cell cytoplasm, nuclei, and bacterial immunostaining input image were shown in Fig 4A. Cytoplasm with nucleus were detected by HCS Cell Mask Deep Red and Hoechst 33258 channel respectively (Fig 4B). Bacterial cells were identified as Alexa 488-staining objects within cytoplasm were specifically detected, and calculated the intensity of them (Fig 4C).

**Fig 5:**
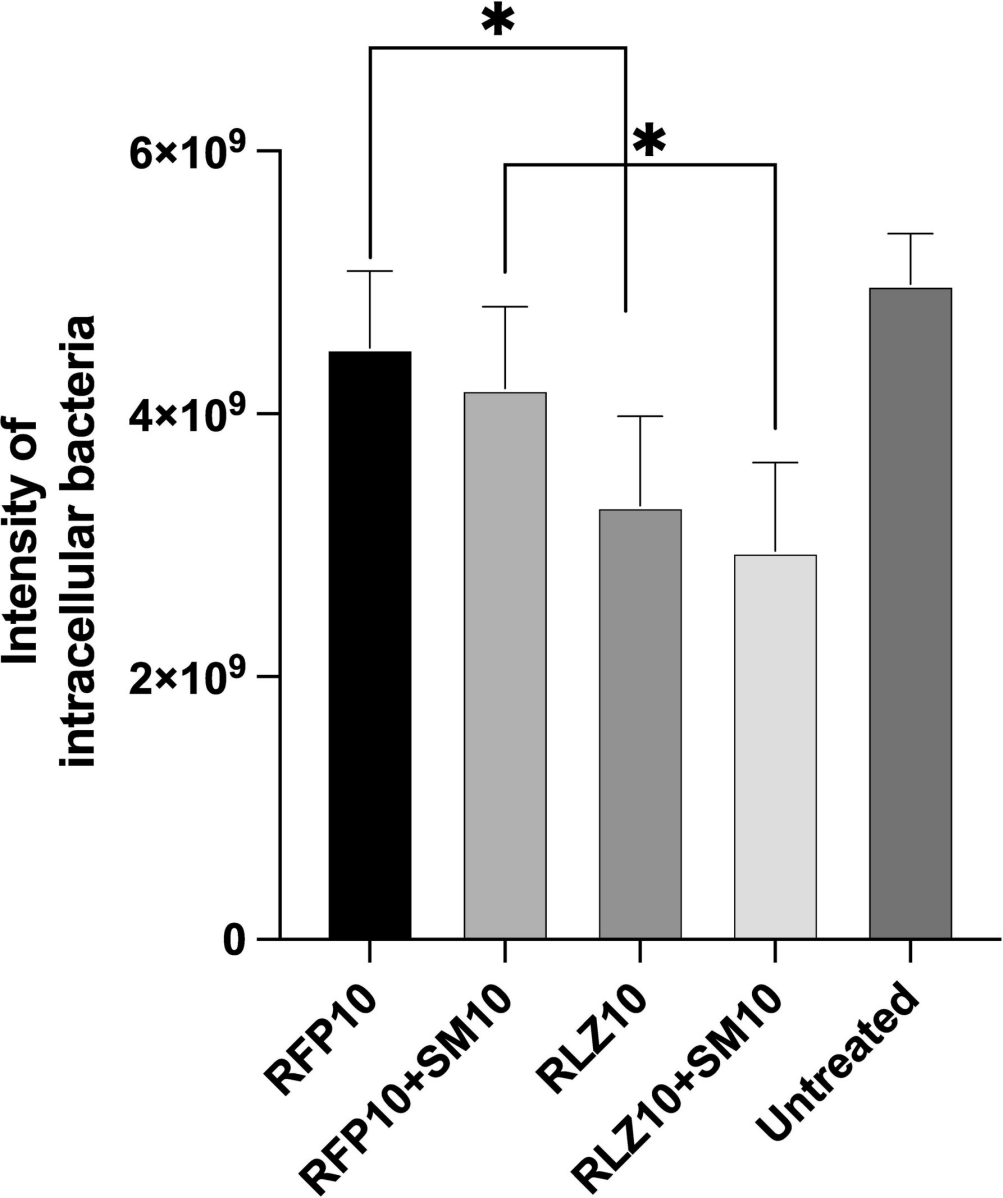
Comparative rifamycin efficacy against intracellular *M*. *ulcerans* with THP-1 human macrophage infection model. The sum of fluorescence intensity (Alexa Flour 488) of intracellular bacterium after 72 hours incubation with RFP10μg/mL, RFP10μg/mL+SM10μg/mL, RLZ 10μg/mL and RLZ 10μg/mL+SM10μg/mL. Data are shown as mean ±SD. *p< 0.05 by unpaired t-test.

Workflow of Harmony software image analysis for intracellular *M*. *ulcerans* showed in Fig 4. In human THP-1 macrophage infection model, intracellular bacterial fluorescent intensity (Alexa 488) of RLZ(10μg/mL) treated wells showed significantly lower than RFP(10μg/mL) (P = 0.0083) (Fig 5). In addition, the intensity of SM/RLZ(10μg/mL) combination treatment showed also significantly lower than SM/RFP(10μg/mL) (P = 0.0077) (Fig 5).

## Discussion

Even novel antimicrobials such as Telacebec to BU are under development, the alternative treatment option might be desired because drug resistance would unexpectedly occur, and once AMR happens, the novel antimicrobial development will take time longer [15].

For a standard treatment for BU, the WHO currently recommends an 8-week combination treatment of rifampin (10 mg/kg once daily) and clarithromycin (7.5 mg/kg twice daily) for the treatment of BU [1, 16]. However, rifampin is a potent CYP3A4 inducer and enhances the metabolism of clarithromycin, which may induce drug resistance [12, 13]. For these reasons, we reevaluated the rifamycin analogue RLZ, which is more effective than rifampin and rifabutin. RLZ was developed for the treatment of *Chlamydia pneumoniae*, *Chlamydia trachomatis*, and *Helicobacter pylori* [17, 18]. RLZ also inhibits the growth of mycobacteria, including *Mycobacterium tuberculosis*, *Mycobacterium avium*, and *Mycobacterium leprae* [19, 20]. Previous studies using *M*. *tuberculosis* and *M*. *avium* demonstrated that RLZ has many advantages over rifampin, including improved drug–drug interactions via CYP450, a longer half-life, higher intracellular activity, and activity against some rifampin-resistant bacteria [21–26]. In addition, RLZ has a lower minimum inhibitory concentration against *M*. *tuberculosis* clinical isolates *in vitro* compared to rifampin and rifabutin [25].

A previous phase II clinical trial analyzed the efficacy of rifampin or RLZ in combination with isoniazid in patients with sputum smear-positive pulmonary TB [25]. Neither rifampin nor RLZ decreased sputum CFUs compared to isoniazid monotherapy. However, this clinical trial was limited in that it only evaluated sputum CFUs and had a short evaluation period of two weeks; therefore, future studies are needed.

Our previous study showed that RLZ is more effective than rifampin in the treatment of a murine *M*. *ulcerans* infection inhibitory model [14]. In the present study, we showed the *in vivo* efficacy of RLZ against advanced *M*. *ulcerans* infection in a murine model, without recurrence following treatment cessation. Importantly, RLZ was sufficiently effective at a low dose of 5mg/kg. THP-1 infection model showed that RLZ showed higher intracellular activity against *M*. *ulcerans* than rifampin. In the streptomycin combination study, RLZ was also significantly more active than rifampicin. In this study, we observed that the CFU numbers were higher at week 9 and week 15 compared to week 6 (RLZ at 5mg/kg) and higher at week 15 compared to week 9 (RLZ at 10mg/kg) is indicative of disease relapse, presumably due to emergence of resistance. Further investigation needs to be performed because detection limits of CFU should be considered. Together, studies suggest that RLZ has therapeutic potential against BU. Based on this study, RLZ might be a valuable alternative for the treatment of BU.

## Supporting information

S1 Table Thickness of hind footpads in *M*. *ulcerans* infected mice treated with or without RLZ. Average of thickness of hind footpads (mean±SD, x10^-2^ mm). Parentheses indicate the number of mice tested.
(XLSX)
